# Leucine-Enriched Essential Amino Acids Augment Muscle Glycogen Content in Rats Seven Days after Eccentric Contraction

**DOI:** 10.3390/nu9101159

**Published:** 2017-10-23

**Authors:** Hiroyuki Kato, Kyoko Miura, Katsuya Suzuki, Makoto Bannai

**Affiliations:** Frontier Research Laboratories, Institute for Innovation, Ajinomoto Co., Inc., Kawasaki, Kanagawa 210-8681, Japan; kyouko_miura@ajinomoto.com (K.M.); katsuya_suzuki@ajinomoto.com (K.S.); makoto_bannai@ajinomoto.com (M.B.)

**Keywords:** muscle damage, leucine-enriched essential amino acids, muscle glycogen, post exercise recovery, eccentric contraction

## Abstract

Eccentric contractions induce muscle damage, which impairs recovery of glycogen and adenosine tri-phosphate (ATP) content over several days. Leucine-enriched essential amino acids (LEAAs) enhance the recovery in muscles that are damaged after eccentric contractions. However, the role of LEAAs in this process remains unclear. We evaluated the content in glycogen and high energy phosphates molecules (phosphocreatine (PCr), adenosine di-phosphate (ADP) and ATP) in rats that were following electrically stimulated eccentric contractions. Muscle glycogen content decreased immediately after the contraction and remained low for the first three days after the stimulation, but increased seven days after the eccentric contraction. LEAAs administration did not change muscle glycogen content during the first three days after the contraction. Interestingly, however, it induced a further increase in muscle glycogen seven days after the stimulation. Contrarily, ATP content decreased immediately after the eccentric contraction, and remained lower for up to seven days after. Additionally, LEAAs administration did not affect the ATP content over the experimental period. Finally, ADP and PCr levels did not significantly change after the contractions or LEAA administration. LEAAs modulate the recovery of glycogen content in muscle after damage-inducing exercise.

## 1. Introduction

Eccentric contractions occur when the muscle lengthens as it contracts and can induce ultrastructural disruption of the skeletal muscle that, in turn, induces several unfavorable symptoms (such as muscle soreness, edema, and muscle dysfunctions) lasting for several days up to several weeks [1]. The long-lasting decline in contractile performance after eccentric contractions is mainly related to the structural disruption of the muscle [2]. However, some reports have pointed to metabolic factors as being responsible for this effect [3,4,5,6,7]. Several studies have showed that eccentric contractions impair the muscle ability to replenish its glycogen content in human study [4,8] and adenosine tri-phosphate (ATP) content in rodent study [6,7] after eccentric exercise. Adenosine tri-phosphate (ATP) is the fundamental source of energy in the cells, and it is utilized for multiple functions (including muscle contractions, maintenance of ion balance, action potential, etc.). Muscle glycogen is the main energy source for moderate-to-high intensity exercises during which it is metabolized to produce ATP. Once glycogen storage is remarkably reduced, the muscular performance at these exercise intensities is severely impaired [9,10,11]. Post-exercise muscle glycogen repletion, usually rapid following exercise [12], is delayed after running a marathon [13]. This happens because of the severe muscle damage due to both concentric and eccentric contraction during the time a marathon occurs (2–6 h) [14]. Therefore, although endurance athletes are generally concerned about the consumption of carbohydrates to replenish muscle glycogen, they should also pay attention to strategies to recover from the muscle damage consequent to their performance to re-accumulate glycogen and regain proper muscle function.

In our previous studies, we found that the administration of a leucine-enriched essential amino acids mixture (LEAAs) enhanced the recovery from muscle damage [15], attenuated muscle soreness, and enhanced muscle protein synthesis [16] after eccentric contractions. Therefore, we hypothesized that LEAAs administration may affect the recovery of glycogen content and the tissue levels of high-energy phosphates molecules, such as phosphocreatine (PCr), adenosine di-phosphate (ADP), and ATP in the damaged muscle by alleviating muscle damage after eccentric contractions. To this end, the present study was designed to examine the effect of LEAAs on muscle glycogen and high-energy phosphates molecules content over a seven-days period after eccentric contractions. As in our previous study [15], we performed our experiments on rat tibialis anterior (TA) muscles, inducing muscle damage through electrically stimulated eccentric contractions.

## 2. Materials and Methods 

This study was approved by the Institutional Animal Care and Use Committee of Ajinomoto Co., Inc. on 30 March 2011 (No. 20111210). All applicable international, national, and institutional guidelines for the care and use of animals were followed.

### 2.1. Animals

Eight-nine weeks old male Sprague–Dawley rats (Charles River Laboratories Japan, Inc., Yokohama, Japan) were housed in a temperature-controlled room on a 12-h light-dark cycle, and provided water and CR-F1 standard commercial chow (Charles River Laboratories Japan Inc., Yokohama, Japan) *ad libitum*.

### 2.2. Experimental Design

The details of the study design were previously described [15]. Briefly, 57 rats were divided into three groups. Sedentary rats received distilled water by oral gavage (Sed, *n* = 19). An equal number of rats additionally underwent electrical stimulation to induce eccentric contraction (EC-Con, *n* = 19), or underwent electrical stimulation and received oral doses of leucine-enriched essential amino acids (1 g/kg BW, EC-AminoL40, *n* = 19) once a day over seven days. Eccentric contraction was induced, as previously described [15,17]. Animals were fasted for 3 h, and electrically stimulated to induce a total of five sets of ten eccentric contractions, each set separated by 1 min of rest. The TA muscle was stimulated percutaneously under inhalation anesthesia with 1.5% isoflurane, using a pair of surface electrodes of a SEN-3301 electrical stimulator (Nihon Kohden Corp., Tokyo, Japan) fitted with an SS-202J isolator (Nihon Kohden Corp., Tokyo, Japan). The muscle was stretched over 900 ms from an ankle position of 45° to 135° using a customized NDH-1 device (Bio Research Center Co., Ltd., Nagoya, Japan), 200 ms after the beginning of the electrical stimulation. Rats were sacrificed immediately after (*n* = 4), or 1, 3, and 7 days (*n* = 5 for each time point) after eccentric contraction and the TA muscles were collected. Tissues were frozen in liquid nitrogen, and stored at −80 °C until analysis.

### 2.3. Leucine-Enriched Essential Amino Acids

The LEAAs mixture (AminoL40) consisted of essential amino acids in the following proportions: histidine, 2%; isoleucine, 11%; leucine, 40%; lysine, 17%; methionine, 3%; phenylalanine, 7%; threonine, 9%; tryptophan, 1%; and, valine, 11%; and, was manufactured by Ajinomoto Co., Inc. (Tokyo, Japan). Except for the elevated proportion of leucine, this mixture contains the ratio of essential amino acids found in whey protein. The AminoL40 mixture was deliberately developed to avoid decreasing the availability of the other essential amino acids (EAAs) while increasing the proportion of leucine [18].

### 2.4. Measurement of High Energy Phosphate Compounds and Muscle Glycogen

For the measurement of high-energy phosphate compounds, a 25-mg muscle sample was homogenized in 350 μL of 0.3 M perchloric acid with 1 mM ethylenediaminetetraacetic acid (EDTA). The homogenate was centrifuged at 13,000 rpm for 5 min at 4 °C. The supernatant was neutralized using 1.5 M KOH with 0.4 M imidazole and 0.3 M KCl (pH 7.6). Following centrifugation at 13,000 rpm for 5 min, at 4 °C, the supernatant was filtered with 0.2 μm mesh filters (Millex-GV, Merck Millipore, Billerica, MA, USA). The filtered supernatants were analyzed using Partisil 10SAX (250 mm × 4.6 mm, GL Sciences Inc., Tokyo, Japan) and a Waters alliance 2690 HPLC separation module (Waters Corporation, Milford, MA, USA) with a Waters 996 PDA Detector (Waters Corporation) set at 254 nm and 210 nm. Samples were eluted with buffer A (0.01 M H_3_PO_4_, pH 2.85) at a flow rate of 0.8 mL/min over 6 min and then, with a linear gradient rising from 0% to 30% Buffer B (0.75 M KH_2_PO_4_, pH 4.40) in 14 min at a flow rate of 1.5 mL/min. The gradient ascended to 100% solvent A at a flow rate of 1.5 mL/min in 0.01 min and was maintained for 9.99 min; then, it was changed to 60% buffer B at a flow rate of 2 mL/min in 0.01 min, and finally switched from 60% buffer B to 100% buffer in 9.99 min at a flow rate of 2 mL/min. Peak areas were quantified by comparison with ADP, ATP, and PCr standards. To determine the glycogen content in the TA, a 30-mg TA muscle sample was hydrolyzed in 2 M HCl, neutralized by adding 2 M NaOH, and assayed for glucose content in the hydrolysate using the Glucose CII Test Wako kit (Wako Pure Chemical Industries, Ltd., Osaka, Japan).

### 2.5. Statistical Analysis

Values are reported as the mean ± the standard error of the mean (SEM). All of the variables were examined by two-way ANOVA, considering treatment and time as factors. When a significant main effect of treatment or interaction was observed, the Tukey’s multiple comparisons test was used to compare groups. Data were analyzed using the GraphPad Prism 6 software (GraphPad Software Inc., San Diego, CA, USA), with *p* < 0.05 considered as significant.

## 3. Results

### 3.1. Muscle Glycogen Content Following Eccentric Contractions

**Figure 1:**
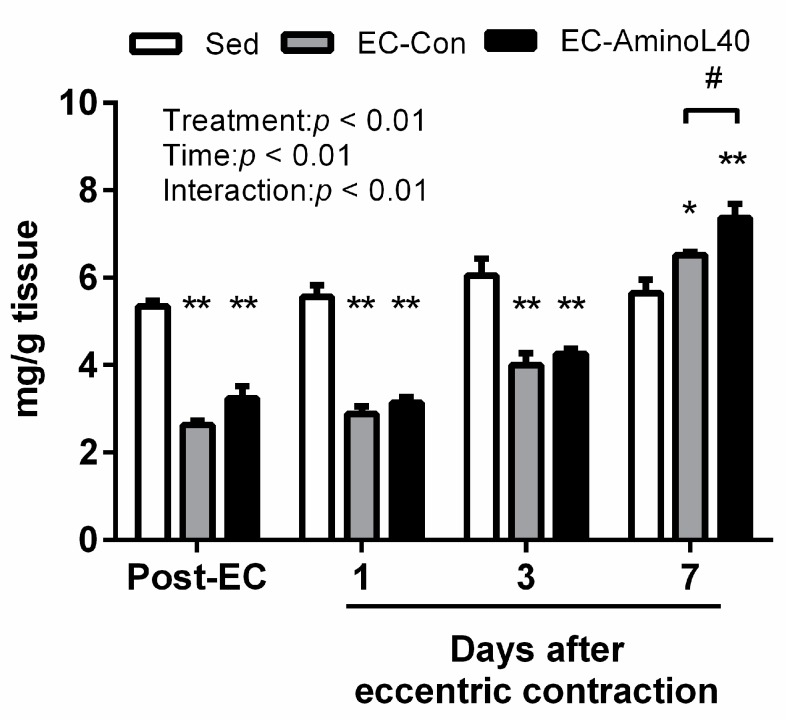
Muscle glycogen content in the tibialis anterior (TA) muscle immediately after eccentric contraction (Post-EC), 1, 3, and 7 days later. Muscle glycogen content was measured in the sedentary group (Sed), and in rats who underwent eccentric contractions and were given water (EC-Con) or a leucine-enriched essential amino acids mixture (EC-AminoL40). Data represent the mean ± SEM (*n* = 4 (Sed) or 5 (EC groups)); * *p* < 0.05 vs. Sed group, ** *p* < 0.01 vs. Sed group, # *p* < 0.05 vs. EC-Con group.

We initially analyzed the glycogen content in the TA muscle following eccentric contractions, and found that it decreased immediately after the contractions and remained low during the following three days, as compared with the Sed group (Figure 1, *p* < 0.01). Interestingly, the muscle glycogen content, decreased after eccentric contractions, was elevated in the EC-Con and EC-AminoL40 groups, compared to the Sed group (*p* < 0.05 and <0.01, respectively) seven days after the contractions; this effect was more remarkable in the EC-AminoL40 group than in the EC-Con one (*p* < 0.05). 

### 3.2. High Energy Phosphate Compounds Content following Eccentric Contraction

**Figure 2:**
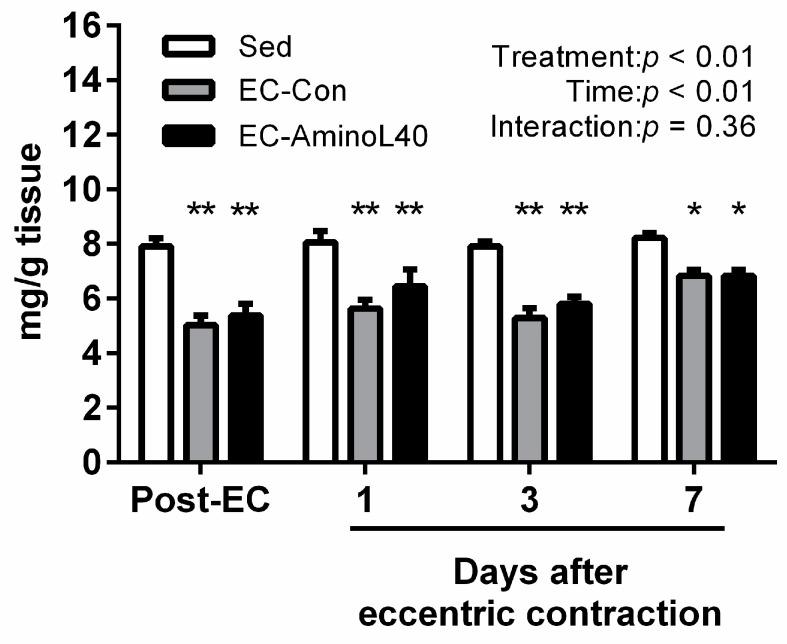
Adenosine tri-phosphate (ATP) content in the tibialis anterior (TA) muscle immediately after eccentric contraction (Post-EC), 1, 3, and 7 days later. ATP was measured in the sedentary group (Sed), and in rats who underwent eccentric contractions and were given water (EC-Con) or a leucine-enriched essential amino acids mixture (EC-AminoL40). Data represent the mean ± SEM (*n* = 4 (Sed) or 5 (EC groups)). * and ** *p* < 0.05 and < 0.01 vs. Sed group at the same time point, respectively.

Next, we analyzed the ATP content in the TA muscle following eccentric contractions, and found it significantly decreased immediately after eccentric contractions and remained lower than in controls up to seven days after (Figure 2, *p* < 0.01 and 0.05 when the Sed group was compared to the EC groups Post-EC, at day one or day three, and at day seven, respectively). In contrast to the changes observed for the muscle glycogen content after eccentric contraction, we did not find any significant difference between the EC-Con and the EC-AminoL40 groups at any time point. 

**Figure 3:**
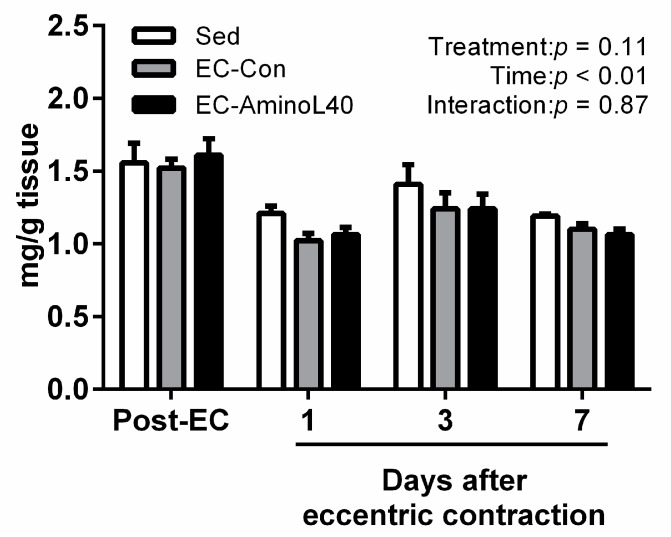
Adenosine di-phosphate (ADP) content in the tibialis anterior (TA) muscle immediately after eccentric contraction (Post-EC), 1, 3, and 7 days later. ADP was measured in the sedentary group (Sed), and in rats who underwent eccentric contractions and were given water (EC-Con) or a leucine-enriched essential amino acids mixture (EC-AminoL40). Data represent the mean ± SEM (*n* = 4 (Sed) or 5 (EC groups)).

**Figure 4:**
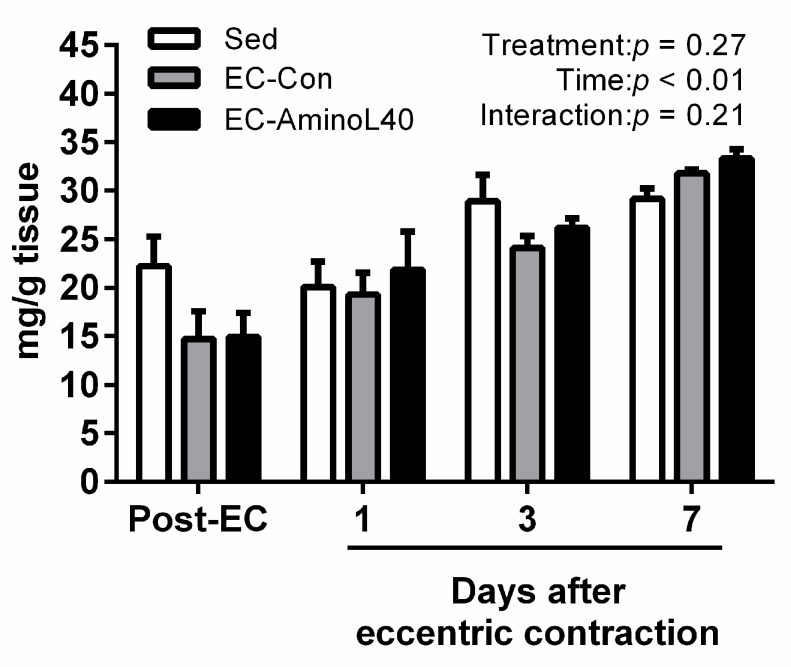
Phosphocreatine (PCr) content in the tibialis anterior (TA) muscle immediately after eccentric contraction (Post-EC), 1, 3, and 7 days later. PCr content was measured in the sedentary group (Sed), and in rats who underwent eccentric contractions and were given water (EC-Con) or a leucine-enriched essential amino acids mixture (EC-AminoL40). Data represent the mean ± SEM (*n* = 4 (Sed) or 5 (EC groups)).

We also measured the ADP and PCr content in the TA muscle after eccentric contractions. However, we did not observe any significant change over the experimental period among the groups analyzed (Figure 3 and Figure 4).

## 4. Discussion

We found that muscle glycogen content decreased immediately after eccentric contractions, and was lower than in controls for the following three days. However, seven days after eccentric contractions, the muscle glycogen content in the EC-Con and EC-AminoL40 groups increased relative to the sedentary group, which had not received eccentric contractions. Earlier studies have reported that a lower muscle glycogen content is detected one and ten days after eccentric exercise, rather than immediately after the exercise [8,19]. The reduced glycogen re-storage might be attributed to two phenomena. First, glucose availability might be lowered in the damaged muscle cells. Several studies have reported the rupturing of the skeletal muscle structure after exhaustive eccentric exercise [2,20], which results in the muscle infiltration of inflammatory cells, including macrophages, leukocytes, and lymphocytes, [21,22], the presence of which is known to increase glucose utilization and lactate production within the muscle [23]. Thus, muscle cells might compete with inflammatory cells in the damaged muscle. Additionally, low glycogen levels might be associated to the reduced activity of glycogen synthase after eccentric exercise [7]. 

Interestingly, administration of LEAAs increased muscle glycogen content, in rats who underwent eccentric contraction, seven days after stimulation, while it did not have any effect (as compared to water) three days after stimulation. Classically, a glycogen-consuming bout of exercise followed by a high carbohydrate diet results in the increase of muscle glycogen to levels above those normally seen in the fed state [24]. This “glycogen supercompensation’’ phenomenon has generally been attributed to the activation of glycogen synthase after exercise and to the increase in glucose uptake and glycogenin level [25]. On the other hand, eccentric exercise decreases insulin-stimulated glucose uptake [26], possibly decreasing the levels of the glucose transporter GLUT4 in the skeletal muscle [27]. Additionally, the possible mechanism of the reduced glucose uptake after eccentric exercise might be associated with an increase in interleukin 6 (IL-6). IL-6 seems to impair the insulin-mediated glucose uptake in the skeletal muscle [28]. Furthermore, as mentioned above, inflammatory cells consume glucose in damaged muscle cells [23]. In our previous study, we found that LEAAs administration interfered with IL-6 expression one day after eccentric contraction, and muscle structural disruption three days after [15]: this might mitigate the disturbance in glucose utilization and contribute to the results obtained in this study. There might be other possible mechanisms through which LEAAs enhance glycogen content following eccentric contraction: LEAAs might modulate the uptake of glucose, which is consequently used for glycogen synthesis, or it might suppress glycogen consumption. Leucine has been reported to increase glycogen synthase activity in muscle cells [29,30]. This finding is supported by the fact that branched-chain amino acids (BCAAs) have been reported to modulate glucose uptake via several mechanisms. First, leucine and isoleucine stimulate glucose transport in skeletal muscle independently of insulin [31,32,33]. Additionally, leucine stimulates insulin secretion, which lowers blood glucose [34,35]: in this regard, it has been found that LEAAs administration decreases blood glucose after strenuous jumping exercise [36]. Therefore, LEAAs might enhance glycogen re-synthesis by increasing the glucose uptake in the skeletal muscle. Alternatively, BCAAs might suppress glycogen consumption. Dietary BCAAs supplementation spared glycogen stored in the skeletal muscle during exercise through the decrease in the activity of the pyruvate dehydrogenase complex [37]. Similarly, de Araujo et al. reported that chronic supplementation with BCAAs increases muscle glycogen concentration in trained rats [38]. In addition, a reduction in the intramuscular ATP content is related to the activation of the branched chain alpha-ketoacid dehydrogenase complex, which is the main enzyme responsible for the oxidation of BCAAs [39]. Therefore, in our experimental model, the reduced ATP content in the muscle might cause an increase in BCAAs catabolism, which leads to the increase in the energy supply. Further studies are needed to confirm this hypothesis and to clarify the mechanism through which LEAAs augment muscle glycogen content after eccentric exercise.

Contrary to the changes in muscle glycogen content, the ATP content of the muscle following eccentric contraction decreased immediately after the stimulation, and did not increase during the investigated time frame (seven days). It remains to be clarified whether, after eccentric contraction, ATP consumption increases or ATP regeneration is impaired. Eccentric contractions impair plasma K^+^ regulation, leading to an elevated ratio of the rise in plasma K^+^ concentration relative to work during contractions [40] and may lead to excitation-contraction failure [2]. In these conditions, ATP consumption would be increased. Additionally, and as mentioned above, the infiltrated inflammatory cells metabolize glucose, inducing energy deficiency in the muscle. Furthermore, during the regeneration phase following muscle damage, increased protein synthesis requires more energy, which is supplied by ATP. On the other hand, eccentric exercise might impair ATP regeneration. Eccentric exercise can lead to muscle damage including dramatic changes in the mitochondrial calcium content and impairment of the respiratory function up to 48 h after exercise [41]. The sustained decline in the ATP content in the damaged muscle may also be due to a lower number of intact mitochondria, the major site of ATP production. The observed decline in the activity of cytochrome C oxidase points towards a lower number of intact mitochondria in the damaged muscle [7]. The mitochondrial calcium handling is impaired by eccentric exercise [42], and may result in a reduced capacity to regenerate ATP. Future studies might unveil the mechanisms causing the decrease in ATP content in damaged muscles. Additionally, there were no significant changes in other high-energy compounds, such as PCr and ADP. Therefore, future studies with a larger sample size are needed to clarify the effect of eccentric exercise on PCr and ADP content.

Although it remains controversial, some studies have reported that protein-based [43,44,45] or BCAAs [46,47] supplements alleviate exercise-induced muscle damage. Other studies, however, indicate that BCAAs supplementation has no effect on the decrease of muscle function and the damage caused by eccentric contraction [48], suggesting that BCAAs alone may not be sufficient to promote recovery from eccentric resistance exercise. Indeed, the ingestion of BCAAs alone increases myofibrillar-muscle protein synthesis (MPS) following exercise, though not maximally because of the lack of other essential amino acids [49]. Contrarily to these data, other studies show that a low dose (3 g) of LEAAs can stimulate muscle protein synthesis equivalently to 20 g of whey protein [18]. Because muscle protein anabolism can modulate the recovery from muscle damage, it will be important, in the future, to understand whether LEAAs are beneficial if administered alone or need to be administered in the context of the whole protein. Additional studies are also necessary to determine the mechanisms responsible for the effects of BCAAs or LEAAs supplementation on muscle soreness and to investigate whether the specific composition of amino acid supplementations changes these effects. 

## 5. Conclusions

We found that eccentric contractions, which induce muscle damage, are associated with a decreased muscle glycogen up to three days after the contraction, and with an increased glycogen content seven days after the stimulation. Daily administration of LEAAs induces a further increase in the glycogen stored in the muscle, as measured seven days after the eccentric contraction. Contrarily, the LEAAs administration did not affect ATP content in the damaged skeletal muscle. These results suggest that LEAAs enhance the recovery of glycogen content after damage-inducing exercise.
